# Analysis of choroidal and central foveal thicknesses in acute anterior uveitis by enhanced-depth imaging optical coherence tomography

**DOI:** 10.1186/s12886-017-0628-7

**Published:** 2017-12-01

**Authors:** Mirinae Kim, Seung Yong Choi, Young-Hoon Park

**Affiliations:** 1 Department of Ophthalmology and Visual Science, Seoul St. Mary’s Hospital, College of Medicine, The Catholic University of Korea, 222 Banpo-daero, Seocho-gu, Seoul, 06591 Korea; 2 Catholic Institute for Visual Science, College of Medicine, The Catholic University of Korea, Seoul, Korea

**Keywords:** Anterior uveitis, Central foveal thickness, Choroidal thickness, Enhanced-depth imaging, Optical coherence tomography

## Abstract

**Background:**

Currently, there are a limited number of reports of structural changes in the retina and choroid in acute anterior uveitis (AAU). The aim of this study was to evaluate choroidal and central foveal thicknesses during episodes of AAU.

**Methods:**

The medical records of 120 patients with AAU and 120 healthy subjects matched for age, sex, and spherical equivalent of refractive error were reviewed. Subjects were divided into group 1 (AAU-affected eyes), 2 (unaffected fellow eyes), and 3 (healthy control eyes).

**Results:**

In the uveitis group, etiologic diagnoses included human leukocyte antigen (HLA)-B27-associated (*n* = 71) and idiopathic (*n* = 49) AAU. The mean subfoveal choroidal thicknesses (SFCTs) in groups 1–3 were 326.7 ± 64.2, 296.1 ± 66.6, and 294.9 ± 41.7 μm, respectively. The corresponding mean central foveal thicknesses (CFTs) were 273.5 ± 29.3, 264.4 ± 24.6, and 263.0 ± 30.8 μm, respectively. The AAU group exhibited a significantly greater SFCT than the control groups (*P* < .001). Relative to the control group, while eyes with idiopathic AAU exhibited a significantly greater CFT, those with HLA-B27-associated AAU exhibited no such significant difference. Anterior chamber cell grade was not associated with SFCT or CFT.

**Conclusions:**

The SFCT increased significantly during AAU. This indicates the importance of OCT examination for detection of subclinical choroidal and retinal changes in all types of AAU.

## Background

Acute anterior uveitis (AAU) is the most common type of uveitis among the general population. The specific cause of uveitis is unclear in approximately 50% of all cases. The most common identifiable etiology—in 40–82% of patients, depending on racial group—is human leukocyte antigen (HLA)-B27-associated uveitis [1–4]. In anterior uveitis, the primary site of inflammation is the anterior chamber, and diagnosis is made on the basis of slit-lamp biomicroscopy findings. Occasionally, cystoid macular edema may be observed (especially in HLA-B27-associated disease); however, this is a more common feature in intermediate, posterior, or pan-uveitis [5]. Most clinicians do not perform optical coherence tomography (OCT) evaluation in case of patients who exhibit normal retinal morphology upon slit-lamp biomicroscopy. For these reasons, choroidal and retinal changes in active anterior uveitis are not well known.

Enhanced-depth imaging (EDI) OCT is a non-invasive technique, which can help easily visualize the choroidal structures. It provides in vivo, cross-sectional, histologic information of the choroid and allows visualization of choroidal vascular structures and measurement of choroidal thickness [6]. In recent years, some studies have used EDI-OCT to evaluate choroidal changes in ocular inflammatory disorders. Some authors have reported that patients with active Vogt–Koyanagi–Harada disease exhibit a markedly thickened choroid [7–9]. In Behçet uveitis, which mainly accompanies panuveitis with retinal vasculitis, a significant change has been reported in the choroidal stroma-to-vessel lumen ratio [10]. In Fuchs’ uveitis syndrome, patients exhibit a relatively thin choroid, which might be the result of the chronic inflammation associated with the disease [11].

Only two studies to date have reported on analysis of choroidal and retinal thicknesses by EDI-OCT in patients with anterior uveitis; however, their reports have presented conflicting results. Basarir et al. [12] evaluated 16 patients with HLA-B27-associated AAU and found that, during active disease period, affected eyes exhibited greater choroidal thickness than unaffected fellow eyes or control eyes; however, central macular thickness was unaffected during the active and convalescent periods. Géhl et al. [13] evaluated 21 patients with active anterior uveitis and found foveal and choroidal thicknesses to be unaffected by the disease; however, the authors did not specify the etiology of anterior uveitis. The discrepancies in results between these two studies are likely because of the small sample size or differences in disease etiology. Moreover, neither study took refractive errors into consideration during analysis.

Therefore, in the present study, we used spectral-domain OCT with EDI technique to comparatively evaluate choroidal and central foveal thicknesses in AAU-affected and unaffected eyes and the eyes of healthy control subjects matched on the basis of age, sex, and spherical equivalent.

## Methods

This retrospective observational study followed the standards for biomedical research laid down in the Declaration of Helsinki, and all protocols were approved by the institutional review board (IRB) of the Catholic University of Korea. Because of the retrospective nature of the study, the IRB waived the requirement for informed patient consent.

This study included 120 patients diagnosed with active AAU. All participants were recruited between April 2011 and December 2016 at Seoul St. Mary’s Hospital in Korea, and a retrospective chart review was conducted. This study only included patients diagnosed with unilateral anterior uveitis with an onset of time of less than 1 week. Diagnosis was made on the basis of presence of inflammatory cells in the anterior chamber and absence of posterior vitreous cells and other features of posterior segment intraocular inflammation. Patients with bilateral uveitis, vitritis, pars planitis, posterior uveitis, panuveitis, or a combination thereof were excluded. In the cases of the contralateral eyes with a previous history of anterior uveitis, we only included the patients with episodes separated by periods of inactivity without treatment over 1 year in duration. Other exclusion criteria were as follows: presence of Vogt–Koyanagi–Harada disease, preexisting macular diseases (e.g., epiretinal membrane, macular hole, or age-related macular degeneration), or pachychoroid pigment epitheliopathy; history of vitreoretinal surgery or refractive surgery; intraocular surgery within 6 months before diagnosis; patients who were using topical, periocular or systemic steroids at the time of image acquisition, and poor image quality due to severe media opacity which can obscure the choroidoscleral border. Healthy subjects matched on the basis of age, sex, and spherical equivalent of refractive error were recruited from among consecutive patients scheduled for routine ocular examination for refractive-error correction at Seoul St. Mary’s Hospital.

Demographic information and comprehensive medical and ophthalmologic history were recorded at initial visit. All subjects underwent ocular examination including evaluation of best-corrected visual acuity (logarithm of the minimum angle of resolution scale, logMAR), non-contact pneumatic tonometry, slit-lamp biomicroscopy, funduscopy, and OCT. Anterior chamber cells were graded from 0 to 4 using a semiquantitative scoring system. Classification and grading of uveitis were performed in accordance with the Standardization of Uveitis Nomenclature (2005, SUN) [14] criteria. In our uveitis clinic, chest radiographs, blood tests (complete blood count, erythrocyte sedimentation rate, C-reactive protein, kidney and liver function tests, antinuclear antibodies, angiotensin-converting enzyme, rheumatoid factor, HLA-B27 and HLA-B51), and a rheumatologic examination were performed on each patient at baseline. Idiopathic cases were defined as cases in which no cause could be found in the comprehensive examination.

**Fig. 1:**
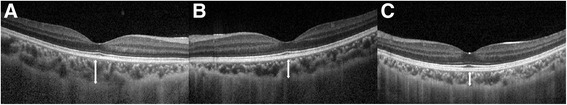
Representative photograph of subfoveal choroidal thickness measurements. Subfoveal choroidal thickness was manually measured at the foveal center using digital calipers provided by the Spectralis OCT software. Subfoveal choroidal thickness was measured by calculating the distance from a hyper-reflective line representing the outer border of the retinal pigment epithelium to the inner edge of the suprachoroidal space, which was represented by a hypo-reflective line on EDI-OCT images. (**a**) eye with acute anterior uveitis, 319 μm (group 1); (**b**) unaffected fellow eye, 258 μm (group 2); (**c**) healthy control eye matched with age, sex and refractive errors, 217 μm (group 3)

Imaging was performed with a Spectralis spectral-domain OCT device (Heidelberg Engineering, Heidelberg, Germany) with software version 6.0. The built-in EDI-OCT feature of the software was used for evaluation of the choroid. From each of the different scan patterns, one horizontal scan with the best image quality was selected for evaluation. Choroidal thickness was manually measured at the foveal center using digital calipers provided by the Spectralis OCT software. Choroidal thickness was measured by calculating the distance from a hyper-reflective line representing the outer border of the retinal pigment epithelium to the inner edge of the suprachoroidal space, which was represented by a hypo-reflective line on EDI-OCT images (Fig. 1) [15]. Central foveal thickness was automatically reported in a modified Early Treatment of Diabetic Retinopathy Study (ETDRS) macular map with a central foveal subfield of 1-mm diameter. To avoid interobserver variation, two experienced independent observers measured the subfoveal choroidal thickness, and the average value was used for statistical analysis.

Categorical data are expressed as absolute numbers, and continuous data as mean ± standard deviation (95% confidence interval). Statistical analysis was performed using the Statistical Package for the Social Sciences for Windows ver. 23.0 (SPSS Inc., Chicago, IL). Normality of data distribution was confirmed by the Kolmogorov–Smirnov test. Comparison of mean values among the three groups was performed by analysis of variance (ANOVA) and the post-hoc Bonferroni test. We used the repeated-measures ANOVA in comparing non-independent samples to consider the within-subject correlation. The relationships between anterior chamber cell grade and central foveal and subfoveal choroidal thicknesses were evaluated by Pearson’s correlation test and regression analysis. The association between central foveal and subfoveal choroidal thicknesses in each group was determined by Pearson’s correlation analysis. *P* values < .05 were considered significant.

## Results

### Demographic and clinical characteristics

A total of 397 patients met the initial inclusion and exclusion criteria of this study. However, only patients with unilateral AAU who had EDI-OCT data available for both eyes were included in the data analysis. Consequently, the study population finally included 120 patients with uveitis. We analyzed 360 eyes of 240 patients: 120 eyes with active AAU (group 1), 120 unaffected fellow eyes (group 2), and 120 healthy control eyes (group 3). There were no significant differences in mean patient age, sex distribution, or refractive error among the three groups.

Table 1 summarizes the demographic and clinical characteristics of patients with uveitis included in this study. The mean age of this group was 44.25 ± 15.3 years, and 66 patients (55.0%) were women. The mean best-corrected visual acuity during the period of active anterior uveitis was 0.22 ± 0.29 logMAR. After resolution of uveitis, the mean best-corrected visual acuity had improved to 0.14 ± 0.23 logMAR. The etiologic diagnoses were as follows: HLA-B27 associated AAU (*n* = 71 [59.1%]); and idiopathic AAU (*n* = 49 [40.9%]). Of the 71 HLA-B27-associated uveitis patients, 48 (67.6%) were confirmed as ankylosing spondylitis. In patients with uveitis, the mean anterior-chamber inflammation grade (determined in accordance with the SUN criteria) was 1.80 ± 0.94, and mean duration of episode of uveitis was 18.2 ± 11.2 days.

**Table 1:** Clinical characteristics of the acute anterior uveitis patients

Variables	Eyes with acute anterior uveitis
Participants (n)	120
Age, years	44.25 ± 15.30
Sex, n (%)	
Female	66 (55.0)
Male	54 (45.0)
SE refractive error (D)	−1.83 ± 2.08
Mean best-corrected visual acuity in uveitic period (logMAR)	0.22 ± 0.29
Mean best-corrected visual acuity after resolution of uveitis (logMAR)	0.14 ± 0.23
Intraocular pressure (mmHg)	13.1 ± 5.1
Etiology of uveitis, n (%)	
HLA-B27-associated	71 (59.1)
Idiopathic	49 (40.9)
Anterior chamber inflammation grade^a^	1.80 ± 0.94
Mean duration of episode of uveitis (days)	18.2 ± 11.2

Data are expressed as mean ± standard deviation (95% confidence interval)

*HLA* Human leukocyte antigen, *logMAR* logarithm of the minimum angle of resolution, *SE* Spherical Equivalent

^a^Anterior chamber inflammation was graded according to the Standardization of Uveitis Nomenclature (2005, SUN)^3^ criteria

### Subfoveal choroidal thickness

**Fig. 2:**
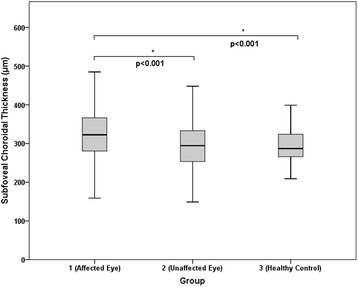
Graph showing the difference in subfoveal choroidal thickness. The mean values of subfoveal choroidal thickness in groups 1–3 were 326.7 ± 64.2, 296.1 ± 66.6, and 294.9 ± 41.7 μm, respectively. Relative to groups 2 and 3 (both, *P* < .001), group 1 exhibited a significantly greater subfoveal choroidal thickness during the acute stage of uveitis. There was no significant difference in subfoveal choroidal thickness between group 2 and 3 (*P* = .998)

Figure 2 presents the subfoveal choroidal thickness measurements of the three groups. The mean values of subfoveal choroidal thickness in groups 1–3 were 326.7 ± 64.2, 296.1 ± 66.6, and 294.9 ± 41.7 μm, respectively (Table 2). Relative to groups 2 and 3 (both, *P* < .001), group 1 exhibited a significantly greater subfoveal choroidal thickness during the acute stage of uveitis. There was no significant difference in subfoveal choroidal thickness between groups 2 and 3 (*P* = .998).

**Table 2:** Comparison of the study groups

Variables	Group 1 (Eyes with acute anterior uveitis)	Group 2 (Unaffected fellow eyes)	Group 3 (Healthy controls)	*P* value
Participants (n)	120	120	120	–
Age, years	44.25 ± 15.30	44.25 ± 15.30	44.47 ± 15.38	.996
Sex, n (%)				
Female	66 (55.0)	66 (55.0)	66 (55.0)	1.000
Male	54 (45.0)	54 (45.0)	54 (45.0)	
SE refractive error (D)	−1.83 ± 2.08	−1.83 ± 2.08	−1.80 ± 2.07	.995
Subfoveal choroidal thickness (μm)	326.7 ± 64.2	296.1 ± 66.6	294.9 ± 41.7	<.001
Central foveal thickness (μm)	273.5 ± 29.3	264.4 ± 24.6	263.0 ± 30.8	.009

Data are expressed as mean ± standard deviation (95% confidence interval)

*SE* Spherical Equivalent

Relative to groups 2 and 3, group 1 exhibited a significantly greatersubfoveal choroidal thickness (P<.001) and central foveal thickness (*P*=.009) during the acute stage ofuveitis

### Central foveal thickness

**Fig. 3:**
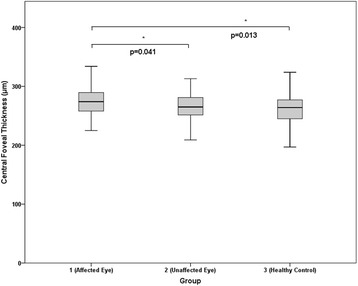
Graph showing the difference in central foveal thickness. The mean values of central foveal thickness in groups 1–3 were 273.5 ± 29.3, 264.4 ± 24.6, and 263.0 ± 30.8 μm, respectively. Relative to groups 2 (*P* = .041) and 3 (*P* = .013), Group 1 exhibited a significantly greater central foveal thickness during the acute stage of uveitis. There was no significant difference in central foveal thickness between group 2 and 3 (*P* = .998)

Figure 3 presents the central foveal thickness measurements of the three groups. The mean values of central foveal thickness in groups 1–3 were 273.5 ± 29.3, 264.4 ± 24.6, and 263.0 ± 30.8 μm, respectively (Table 2). Relative to groups 2 (*P* = .041) and 3 (*P* = .013), group 1 exhibited a significantly greater central foveal thickness during the acute stage of uveitis. There was no significant difference in central foveal thickness between groups 2 and 3 (*P* = .998).

Anterior-chamber cell grade was not significantly associated with subfoveal choroidal thickness (rho = 0.101; *P* = .273) or central foveal thickness (rho = −0.072; *P* = .436). No significant correlation was observed between retinal and choroidal thicknesses in any of the three groups (rho = 0.149, *P* = .235 [group 1]; rho = −0.036, *P* = .695 [group 2]; and rho = 0.179, *P* = .051 [group 3]; Pearson’s correlation test).

### Subgroup analysis

In subgroup analyses, we analyzed the subfoveal choroidal and central foveal thicknesses in patients with HLA-B27-associated and idiopathic anterior uveitis (*n* = 71 and 49, respectively).

**Fig. 4:**
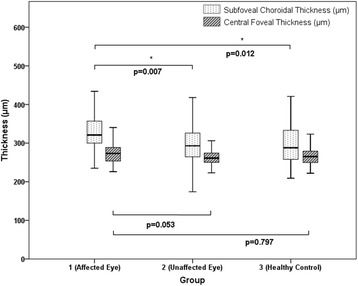
Graph showing the subfoveal choroidal thickness and central foveal thickness in HLA-B27 associated uveitis group (*n* = 71). In subgroup analysis, we analyzed the subfoveal choroidal thickness and central foveal thickness in HLA-B27 associated anterior uveitis patients (*n* = 71). Relative to groups 2 (*P* = .007) and 3 (*P* = .012), the subgroup of patients with HLA-B27-associated anterior uveitis exhibited a significantly greater subfoveal choroidal thickness during the acute stage of the disease. There was no significant difference in the central foveal thickness

Relative to groups 2 (*P* = .007) and 3 (*P* = .012), the subgroup of patients with HLA-B27-associated anterior uveitis exhibited a significantly greater subfoveal choroidal thickness during the acute stage of the disease. There was no significant difference in central foveal thickness between this subgroup and groups 2 and 3 (Fig. 4).

**Fig. 5:**
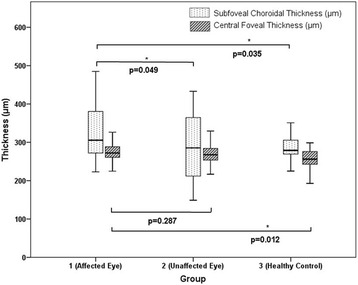
Graph showing the subfoveal choroidal thickness and central foveal thickness in idiopathic uveitis group (*n* = 49). In subgroup analysis, we analyzed the subfoveal choroidal thickness and central foveal thickness in idiopathic anterior uveitis patients (*n* = 44). Relative to groups 2 (*P* = .049) and 3 (*P* = .035), patients with idiopathic anterior uveitis exhibited a significantly greater subfoveal choroidal thickness during the acute stage of the disease. This subgroup exhibited a greater central foveal thickness than groups 2 (*P* = .287; insignificant difference) and 3 (*P* = .012)

Relative to groups 2 (*P* = .049) and 3 (*P* = .035), patients with idiopathic anterior uveitis exhibited a significantly greater subfoveal choroidal thickness during the acute stage of the disease. This subgroup exhibited a greater central foveal thickness than groups 2 (*P* = .287; insignificant difference) and 3 (*P* = .012) (Fig. 5)**.**

## Discussion

In this study, we observed significant increases in subfoveal choroidal and central foveal thicknesses during the active period of AAU. This relationship was evident even in eyes with only mild anterior inflammation. While cystoid macular edema may be observed in eyes with anterior uveitis, it has been reported in only a minority of patients (4–9% of AAU cases) [5, 16]. Most clinicians do not perform OCT evaluation in case of patients who present with normal retinal morphology upon slit-lamp biomicroscopy. In fact, in the present study, only 30.2% of the initial participants (120 of 397 patients with anterior uveitis) had undergone EDI-OCT. Subclinical macular edema might be easily misdiagnosed on the basis of biomicroscopy findings. Previously, Shulman et al. [17] had reported that some degree of macular edema or peripapillary retinal nerve fiber layer thickening can be detected even in mild anterior uveitis. In patients with uveitis, OCT is as effective as fluorescein angiography at detecting cystoid macular edema; the sensitivity and specificity of OCT for detection of cystoid macular edema have been reported to be 96% and 100%, respectively [18]. This, together with the present findings, indicates the importance of OCT examination in all types of active anterior uveitis.

It is widely known that the choroid is influenced by ocular inflammatory conditions, especially in posterior uveitis [7, 19–21]. Several authors have suggested increased vascular permeability in the posterior segment and choroidal effusion as the mechanisms of choroidal thickening during ocular inflammation. Furthermore, in case of mild anterior chamber inflammation, changes in choroidal and central foveal thicknesses are associated with the breakdown of the blood–retina barrier [22, 23]. The present results suggest that even mild anterior chamber inflammation might cause some degree of posterior segment changes.

Our study methodology might call into question the reliability and repeatability of manual measurement of choroidal thickness using EDI-OCT images. Rahman et al. [24] reported intra- and interobserver coefficients of repeatability of 23 and 32 μm, respectively. In the present study, two experienced independent observers performed subfoveal choroidal thickness measurements, and the average value was used for statistical analysis. The mean difference in subfoveal choroidal thickness measurements between the 2 observers was 0.8 um (*P* = .625, paired-t test).

According to previous studies, the mean subfoveal choroidal thickness in healthy subjects, measured by EDI-OCT, ranges from 287 to 332 μm [24, 25]. In the present study population, the mean values of subfoveal choroidal thickness in eyes with AAU, unaffected eyes, and healthy control eyes were 326.7 ± 64.2, 296.1 ± 66.6, and 294.9 ± 41.7 μm. Although the AAU group exhibited significant choroidal thickening, it could be considered as being within the normal range if not for consideration of the unaffected eye. These findings suggest the need for bilateral OCT evaluation in patients with anterior uveitis. Additionally, because the choroid is a frequent target for intraocular inflammation, in vivo imaging of the choroidal vasculature by EDI-OCT might be helpful in evaluation of specific disease processes [26].

Géhl et al. [13] conducted a case-control study of 21 patients with anterior uveitis and 23 patients with intermediate uveitis. While the authors found no significant difference in central subfoveal thickness between patients with anterior uveitis and control subjects, the former group exhibited a substantially higher retinal thickness in ETDRS 3-mm and 6-mm-diameter zones. Castellano et al. [27] reported that retinal thickness is strongly associated with iridocyclitis and that it decreases after treatment. In agreement with previous findings, the present results confirmed an increase in retinal thickness during active AAU.

The results of subgroup analyses revealed that, relative to the control group measurements, subfoveal choroidal thickness was significantly greater during the acute stage of uveitis, regardless of the etiologic diagnosis. In contrast, relative to the control group measurements, while central foveal thickness was significantly greater in the idiopathic AAU subgroup, the difference was not significant in the HLA-B27-associated AAU subgroup. This result is consistent with that of a previous report by Basarir et al. [12] who evaluated 16 patients with HLA-B27-associated AAU and found that, while affected eyes exhibited greater choroidal thickness than the fellow unaffected eyes, there was no substantial difference in central macular thickness. Although posterior segment involvement in HLA-B27-associated AAU has been reported [28, 29], it is under-recognized. The exact mechanism of HLA-B27-associated uveitis is not well known; however, our findings suggest that immune-mediated responses accompanying the uveitic episode mainly affect the choroidal vasculature in HLA-B27-associated AAU. In contrast, in idiopathic uveitis, there is no preference for choroidal or retinal vasculature.

Our study has some limitations. First, our results may be affected by the lack of follow-up measurements during the chronic or resolution phase. Second, some authors have reported a significant circadian (diurnal) variation—of approximately 20–30 μm—in choroidal thickness measurements by OCT [30–32]. The participants of our study underwent OCT at various times of the day, and this might have affected our results. Third, we only evaluated the 1-mm-diameter central foveal subfield in a modified ETDRS map; there might have been unknown changes in the 3-mm- or 6-mm-diameter subfield. And, we used the automated segmentation of SD-OCT for central foveal thickness. There is a possibility of segmentation errors, especially in eyes with severe inflammation. These limitations should be addressed in future studies.

## Conclusions

In summary, during active inflammation, eyes with AAU exhibited greater subfoveal choroidal and central foveal thicknesses than unaffected fellow eyes and healthy control eyes. However, retinal thickening may or may not be evident depending on the etiology of anterior uveitis. These results suggest the importance of OCT examination for detection of subclinical choroidal and retinal changes in all types of active anterior uveitis. Further large-scale studies with long-term follow-up data are necessary to confirm our results.

## Abbreviations

AAU: Acute anterior uveitis
CFT: Central foveal thickness
EDI: Enhanced-depth imaging
HLA: human leukocyte antigen
logMAR: logarithm of the minimum angle of resolution
OCT: Optical coherence tomography
SE: Spherical equivalent
SFCT: Subfoveal choroidal thickness
